# Analysis of *PAH* Genetic Variation and Phenotypic Diversity in the PAHvdb

**DOI:** 10.3390/ijms27156772

**Published:** 2026-07-29

**Authors:** Asylulan Amirgazin, Bakhyt Mambetpayeva, Assel Akhmetova, Alma Kairzhanova, Alexandr Shevtsov

**Affiliations:** 1 Laboratory of Applied Genetics, National Center for Biotechnology, Astana 010000, Kazakhstan; asylulan0894@gmail.com (A.A.); kairzhanova@biocenter.kz (A.K.); ncbshevtsov@gmail.com (A.S.); 2 Medical Genetics and Molecular Biology Department, NCJSC “Astana Medical University”, Astana 010000, Kazakhstan; mambetpaeva.b@amu.kz

**Keywords:** phenylketonuria, hyperphenylalaninemia, *PAH* gene, PAHvdb, variant spectrum

## Abstract

The Phenylalanine Hydroxylase Gene Locus-Specific Database and BIOPKU are public databases used to track genotypes in hyperphenylalaninemia. However, the rapidly expanding databases and evolving nomenclature standards require a comprehensive characterization of the current variant landscape. We performed a descriptive statistical analysis of *PAH* variant allocations, allele frequencies (AF%), and phenotypic annotations using PAHvdb database. Over the period 2022–2026, the PAHvdb expanded by 169.26%. Of the 3409 variants studied, 75.68% of the phenotypes were not assigned. Splicing-related mutations comprised approximately 4.37% of the database, with classic phenylketonuria phenotypes represented in both donor–acceptor and splice-region categories. Only 3.84% of the variants had enzymatic activity (EA%) data, which offer the most direct biochemical evidence for genotype–phenotype inference. The strong Spearman’s rank correlation coefficient (ρ) between EA% and APV (ρ = 0.745, *p* < 0.001) was consistent with the established relationship between residual PAH EA% and the severity of metabolic phenotype. The AF% distribution was markedly asymmetric and dominated by a small number of alleles that were most frequently reported in the database. These findings highlight the increasing clinical reliance on these databases. Our analysis highlights a heterogeneity of rare, uncharacterized variants of uncertain significance and underscores the need for unified, systematic reviews to standardize nomenclature and resolve phenotype–genotype discrepancies.

## 1. Introduction

Hyperphenylalaninemia (HPA) is a group of autosomal recessive disorders characterized by the accumulation of phenylalanine (Phe) in the body caused by insufficient activity of the L-phenylalanine hydroxylase enzyme (PAH; EC 1.14.16.1) [1]. Toxically high concentrations of Phe can cause the formation of phenylpyruvate and phenylketone bodies, monoamine neurotransmitter deficiency, hypotyrosinemia, and other amino acid metabolism disorders [2]. Lack of treatment leads to disruption of dendritic growth and synaptic connectivity in neurons of the brain’s white and gray matter, disruption of myelination, formation of amyloid-like fibrils, and changes in glucose metabolism [2]. These metabolic and neurological pathologies are associated with a variety of symptoms: severe mental retardation, developmental delay, epilepsy, light pigmentation, eczema, musty odor, blindness, and memory impairment, as well as behavioral, psychiatric, and movement disorders [2].

Phenylketonuria (PKU), or phenylalanine hydroxylase deficiency (PAHD), is a disease caused by biallelic pathogenic mutations in the *PAH* gene [3]. PAHD/PKU accounts for the vast majority of HPA cases (~98%) [4]. The remaining HPA cases (~2%) are caused by pathogenic mutations (variants) in the genes encoding tetrahydrobiopterin (BH_4_) synthesis and regeneration, as well as in the gene encoding the DNAJC12 chaperone [5]. The *PAH* gene is located in the region 12q22-q24.2 on chromosome 12 and is 171 kb in length (including untranslated regions). The gene consists of 13 exons and 12 introns and encodes a 452-amino acid (51.8 kDa) monomeric protein with three domains: N-terminal regulatory domain, central catalytic domain, and C-terminal oligomerization domain. *PAH* is a homotetrameric iron-containing monooxygenase enzyme that catalyzes the hydroxylation of L-phenylalanine to form L-tyrosine. *PAH* activity requires molecular oxygen, iron ions, and BH_4_ [6].

The determining factor in the phenotypic manifestation of HPA is the residual enzymatic activity of *PAH*. Depending on the initial Phe level in the blood, HPA phenotypes are classified as follows: mild hyperphenylalaninemia (MHP)—120–600 µmol/L (2–6 mg/dL); mild PKU (mPKU)—600–1200 µmol/L (10–20 mg/dL); and classic PKU (cPKU)—>1200 µmol/L (>20 mg/dL) [7]. In most countries, HPA diagnosis is included in neonatal screening based on the determination of Phe levels in dried blood spots using tandem mass spectrometry [8]. Furthermore, in patients with Phe levels above normal (35–120 µmol/L, <2 mg/dL), BH_4_ deficiencies, DNAJC12, and liver diseases are excluded, as they require different treatment strategies [8]. Final differentiation and confirmation of the diagnosis is carried out by genotyping of genes associated with HPA, the most popular of which is targeted next-generation sequencing (NGS) of the *PAH* gene [9,10]. Determining the patient’s genotype allows prediction of the metabolic phenotype and selection of an immediate treatment strategy [3].

HPA treatment maintained from the first days after birth prevents severe clinical consequences, and neurocognitive functions remain suboptimal on average [2,11]. Patients with insufficient or zero PAH activity (cPKU) are prescribed a strict lifelong diet with a limited amount of Phe. Defects in BH_4_ metabolism respond to lifelong pharmacological treatment with sapropterin dihydrochloride, a synthetic analog of BH_4_, as do a subset of patients with PKU (~20–30%) [12,13]. *PAH* genotypes with high residual enzymatic activity (EA%) have a higher probability of responding to BH_4_, due to stabilization of the enzyme homotetramer [14,15]. The accuracy of predicting sensitivity to BH_4_ is ~71% [16]. However, the vast majority of known *PAH* variants are rare, poorly annotated, or associated with the classical (cPKU) phenotype. As a result, *PAH* genotyping is primarily useful for identifying patients unlikely to respond to sapropterin, rather than for reliably predicting who will respond [17].

New variants are regularly identified during genotyping of patients from various populations, as the frequency and spectrum of *PAH* mutations vary among different countries and may also differ within a single country [10]. The publicly available databases for hyperphenylalaninemia and related metabolic disorders, including the Phenylalanine Hydroxylase Gene Locus-Specific Database (PAHvdb) and the BIOPKU genotypes database, are hosted concurrently at www.biopku.org. These databases collect anonymized data on the genotypes of patients with HPA and corresponding phenotypes. Based on these data, genotype-based phenotype prediction was possible for 99.2% of cPKU cases, 46.2% of mPKU cases, and 89.5% of MHP cases [3]. The genotype–phenotype prediction values (GPVs) showed high statistical significance relative to baseline blood Phe levels and the response to BH_4_ [3]. Developing and accurately maintaining the Phenylalanine Hydroxylase Gene Locus-Specific Gene Database (like PAHvdb/BIOPKU) is crucial for clinical decision-making. Using these data helps diagnose PKU, predict disease severity based on genotypes, and determine patients’ response to therapy, such as BH_4_.

In March 2026, the National PKU Alliance (NPKUA) acquired the BIOPKU database and its associated PAHvdb to expand and further support this research resource. A previous descriptive review of PAHvdb was conducted in 2022, when the database contained ~1285 unique *PAH* alleles; however, it has grown more than 1.5-fold since then. Therefore, an expanded descriptive analysis is required to update current knowledge of variant and phenotypic diversity associated with phenylketonuria.

The aim of the current study was to provide an updated analytical review of the PAHvdb database and characterize the expanding mutational spectrum of the *PAH* gene. Using simple informatics methods, we analyzed the types, locations, frequencies, and phenotypic manifestations of known *PAH* variants. The results shed light on the current state of the database, facilitate its accurate maintenance, and improve understanding of the genetic basis of phenylketonuria.

## 2. Results

**Figure 1:**
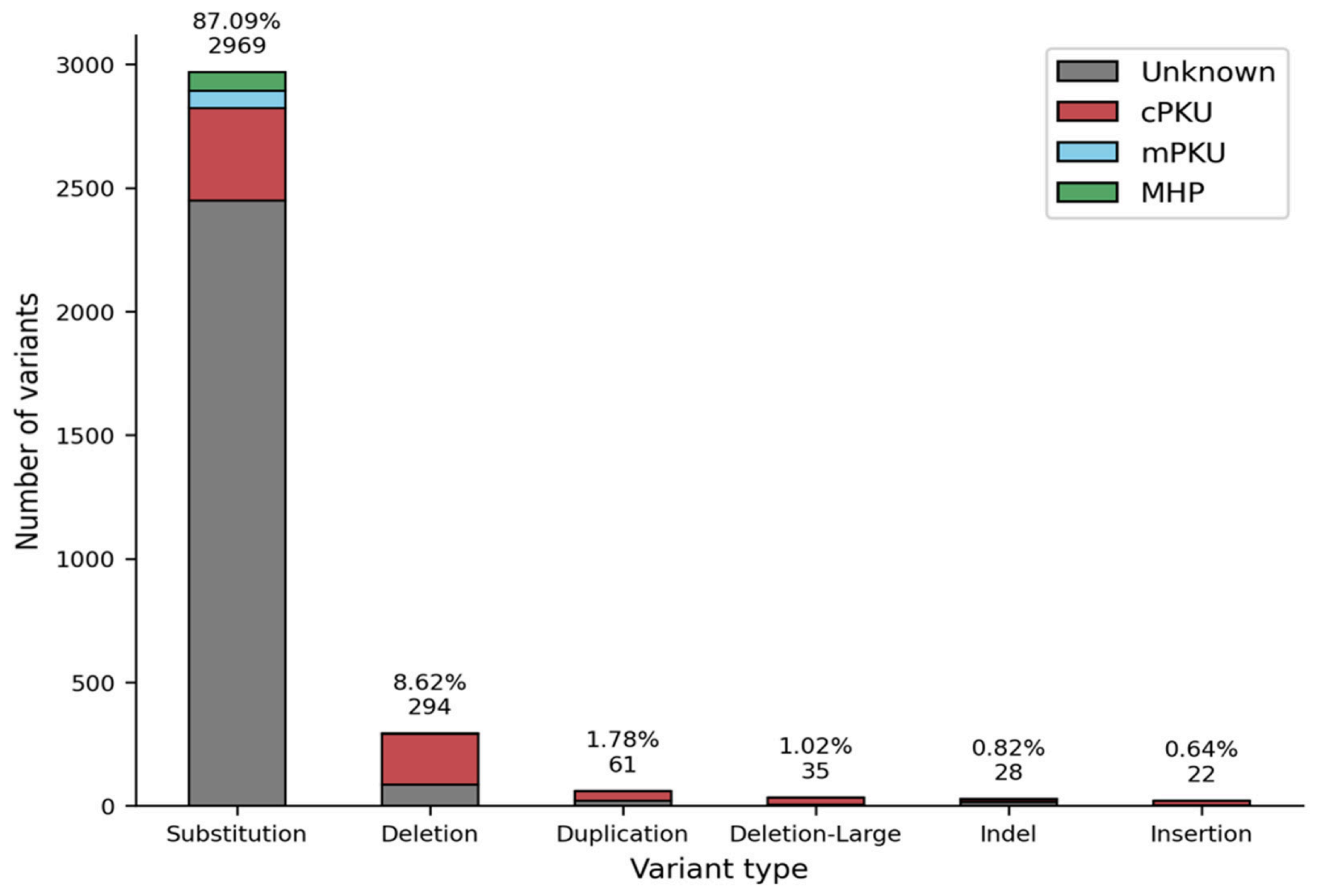
Distribution of *PAH* gene mutation types and associated phenotypes. Each bar represents a different variant type (*X*-axis), and colors represent phenotype diversity, with the number of variants and the percentage of the total 3409 variants analyzed.

For all 3409 analyzed variants, information on the mutation type, effect, and location was available. The vast majority of mutations identified in the *PAH* gene were nucleotide substitutions (87.09%; *n* = 2969). Although the phenotype was unknown for most nucleotide substitutions (82.5%, *n* = 2449), the proportion of substitutions associated with the cPKU phenotype was the highest among mutation types—12.6% (374 variants). Deletions accounted for the second-largest proportion of variants, at 8.62% (*n* = 294). Moreover, the cPKU phenotype was related to deletion variants, which accounted for 69.7% (205 variants). The remaining variant types collectively accounted for 4.29% of the total (Figure 1, Table S2). Among these, duplications accounted for 1.78% (*n* = 61), while large deletions (Deletion—Large) accounted for 1.02% (*n* = 35). The smaller proportions accounted for indels (0.82%; *n* = 28) and insertions (0.64%; *n* = 22).

**Figure 2:**
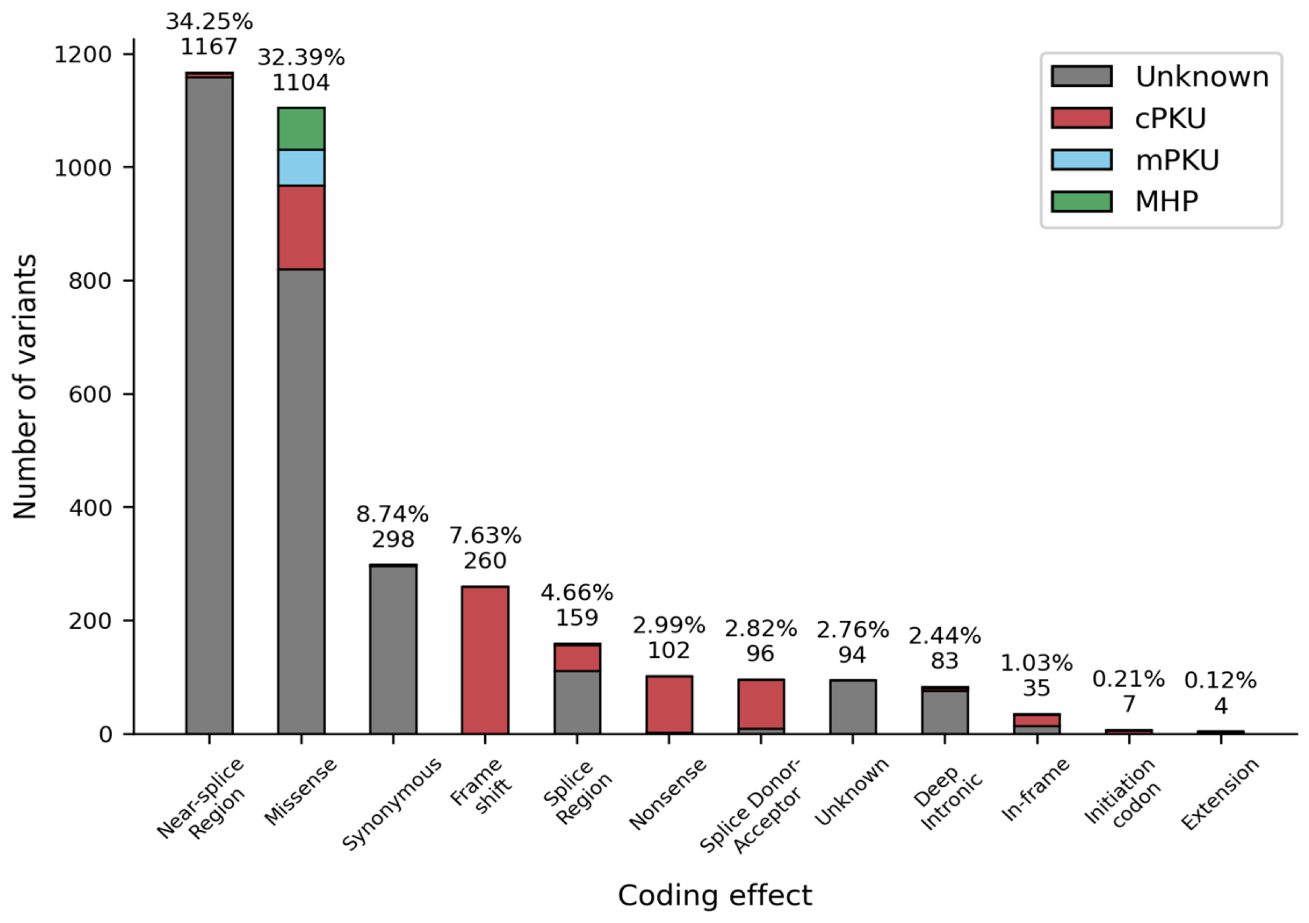
Mutational landscape and phenotypic distribution of *PAH* variants. Various *PAH* genetic mutation effects are shown on the *X*-axis, with different bar colors showing associated phenotypes, the number of variants, and the percentage of the total 3409 variants analyzed.

Among variant effect annotations, near-splice region mutations represented the most frequent category, accounting for 34.25% (*n* = 1167) of the total number of variants. Missense mutations accounted for a roughly comparable proportion, at 32.39% (*n* = 1104). However, these two categories differed from other effect types (Figure 2, Table S3).

Despite the large number of mutations in the near-splice region category, 99.23% were of unknown phenotype (*n* = 1158). However, cases with identified phenotypes of cPKU (*n* = 7), mPKU (*n* = 1), and MHP (*n* = 1) were still present. Most missense mutations also comprised variants with an unknown phenotype (74.28%, *n* = 820). However, this category exhibited the widest diversity of confirmed phenotypes (cPKU: 13.32%, mPKU: 5.8%, MHP: 6.61%). The proportion of the frameshift variant effects group was 7.63% (*n* = 260) and was represented only by the cPKU phenotype. Other categories predominantly causing cPKU (>90% of variants) were nonsense (2.99%, *n* = 102) and splice donor–acceptor (2.82%, *n* = 96). Mutations affecting splice sites (splice region, 4.66%, *n* = 159) were also associated with cPKU (28.3%).

Synonymous mutations were observed in 8.74% (*n* = 298); two variants were associated with the cPKU and mPKU phenotypes. The remaining 296 variants of synonymous variants (99.33%) represented an unknown phenotype. Deep intronic mutations, potentially affecting splicing regulation, accounted for 2.44% (*n* = 83). Of these, four variants were associated with cPKU, two variants with mPKU, and one variant with MHP. The least represented categories were in-frame mutations (1.03%, *n* = 35), mutations altering the initiation codon (0.21%, *n* = 7), and mutations causing protein extension via stop codon disruption (0.12%, *n* = 4). Mutations with an unknown effect accounted for 2.76% (*n* = 95).

**Figure 3:**
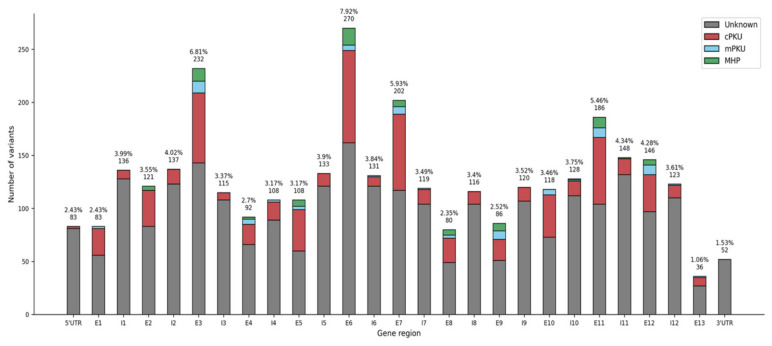
Regional distribution of *PAH* mutations and their corresponding clinical phenotypes. Total variant counts plotted across the 13 exons and flanking intronic regions of the *PAH* gene. The relative proportions of classical PKU (cPKU), mild PKU (mPKU), and mild hyperphenylalaninemia (MHP) phenotypes associated with variants in each structural region are shown in different colors.

The majority of identified variants were localized within the catalytic domain of the enzyme (*n* = 1178, 34.55%), followed by the regulatory domain (*n* = 420, 12.32%) and oligomerization domain (*n* = 138, 4.04%). The most affected regions of the *PAH* gene were exon 6 (*n* = 270, 7.92%), exon 7 (*n* = 202, 5.93%), exon 3 (*n* = 232, 6.81%), and exon 11 (*n* = 186, 5.46%) (Figure 3). They also had the highest number of variants associated with the cPKU phenotype compared to other regions (87 variants—32.22%, 72 variants—35.64%, 66 variants—28.45%, and 63 variants—33.87%, respectively). However, in terms of the percentage of cPKU-causing variants in an exon, exon 5 had the highest percentage (*n* = 39, 36.11%), followed by exon 7 and exon 10 (*n* = 40, 33.9%). The remaining regions of the *PAH* gene had fewer than 150 variants (Figure 3, Table S5). The lowest variant frequencies were observed within the 3′UTR (*n* = 52, 1.53%) and exon 13 (*n* = 36, 1.06%).

Thus, 1760 (51.63%) variants located in the coding part of the gene (exons) were represented by the following phenotypes: cPKU—15.58% (*n* = 531), mPKU—1.99% (*n* = 68), MHP—2.14% (*n* = 73), and unknown phenotype—31.92% (*n* = 1088). While 1649 (48.37%) variants located in the non-coding part of the gene (introns and UTR) were represented by cPKU—4.37% (*n* = 149), mPKU—0.18% (*n* = 6), MHP—0.06% (*n* = 2), and unknown phenotype—43.76% (*n* = 1492). Variants located in introns and associated with the cPKU phenotype belonged to the following categories: splice donor–acceptor (58.38%, *n* = 87), splice region (30.2%, *n* = 45), near-splice region (4.69%, *n* = 7), deep intronic (2.68%, *n* = 4), and frameshift (2.68%, *n* = 4). Their proportions ranged from 5.88% (intron 1) to 15.74% (intron 4). The proportions of variants of unknown phenotype located in exons ranged from 55.56% (exon 5) to 75% (exon 13). These variants carried no APVs; a total of 334 variants carried AF% ranging from 0.002 to 0.04, while 754 lacked associated AF% values. For variants located in exons and associated with cPKU (*n* = 531), AF% ranged from 0.002 to 16.32 for 363 variants, and AF% values were missing for 168 variants.

Of the 3409 variants, APVs were available for 829 variants (24.31%). Among the 829 variants with available APVs, the cPKU phenotype was predominant at 82.02% (*n* = 680), followed by mPKU, 8.92% (*n* = 74), and MHP, 9.04% (*n* = 75). Thus, the PAHvdb database included 75.68% of variants (*n* = 2580) of unknown phenotype, 19.94% of cPKU variants, 2.17% of mPKU variants, and 2.20% of MHP phenotype variants.

Information on enzyme activity (EA%) was available for 131 variants (3.84%) out of 3409. However, only 97 of these variants contained APVs. The sensitivity analysis showed that the correlation between enzymatic activity and phenotype was unchanged when the two variants with reported EA% values exceeding 100% were included or excluded. Including these observations yielded a Spearman correlation coefficient of ρ = 0.792 (*p* = 1.51 × 10^−22^), whereas excluding them resulted in ρ = 0.796 (*p* = 2.03 × 10^−22^). Therefore, despite the limited number of data, EA% was positively correlated with APVs.

**Figure 4:**
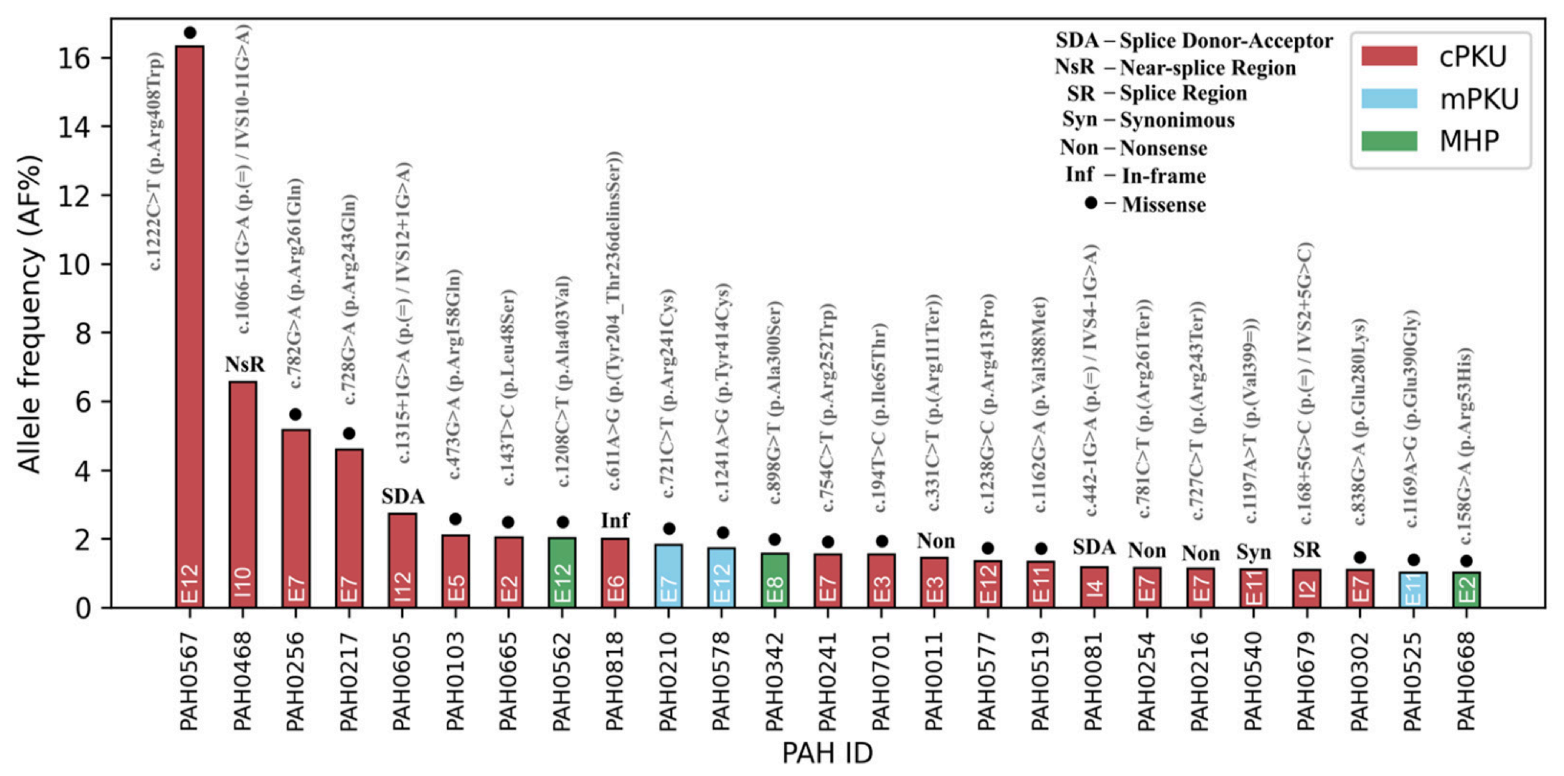
Distribution of *PAH* variants with an allele frequency >1%. The barplot shows *PAH* variants (*X*-axis) and their locations, with associated phenotypes shown in different colors. Individual variants are explicitly labeled to indicate their predicted functional mutation effects. The *Y*-axis represents the raw or relative allele frequency.

Allele frequency (AF%) values were available for 996 variants (29.21%) out of 3409 variants. To prevent statistical bias in prevalence estimates, variants with unspecified or missing APVs and AF% values (“NaN” or “Unknown”) were systematically excluded from the analysis. The filtered database included 624 variants, of which 25 alleles had AF% > 1 (Figure 4, Table S6). All 25 *PAH* alleles were caused by nucleotide substitutions, and the corresponding AF% ranged from 1.02% to 16.32%. The majority of them were missense mutations (16 alleles). Other *PAH* genetic mutation effects accounted for nonsense (3 alleles), splice donor–acceptor (2 alleles), in-frame (1 allele), near-splice region (1 allele), splice region (1 allele), and synonymous (1 allele). In total, missense mutations accounted for 46.37% (16 alleles), and other mutations accounted for 18.48% (9 alleles).

Most represented in the database alleles also included variants located in non-coding regions, specifically at the edges of introns at splice sites. For example, the second most frequently reported variant was a nucleotide substitution belonging to the near-splice region category—PAH0468 (c.1066-11G>A). Moreover, the mutation was located 11 bp from the canonical splice site (CSS). Variants PAH0605, PAH0081, and PAH0679 were also frequently observed among the dataset (AF% = 1.11–2.74) and were associated with the cPKU phenotype. Variants PAH0605 and PAH0081 belonged to the splice donor–acceptor category, and identified mutations were localized within the core CSS (within 1 bp). Variant PAH0679 belonged to the splice region category and was localized 5 bp from the CSS. Variant PAH0540 was also unique, as it was caused by a synonymous mutation located 3 bp from the CSS [18]. This is the only variant in the synonymous category associated with cPKU.

As a result, the three most frequent alleles among all variants within the current dataset coincided with those most observed in the cPKU phenotype: PAH0567 [c.1222C>T (p.Arg408Trp)] with a frequency of 16.32%, PAH0468 [c.1066-11G>A (p.(=)/IVS10-11G>A)] with a frequency of 6.56%, and PAH0256 [c.782G>A (p.Arg261Gln)] with a frequency of 5.18%. The three most frequent alleles in the dataset of the mPKU phenotype were PAH0210 [c.721C>T (p.Arg241Cys)] with a frequency of 1.84%, PAH0578 [c.1241A>G (p.Tyr414Cys)] with a frequency of 1.72%, and PAH0525 [c.1169A>G (p.Glu390Gly)] with a frequency of 1.02%. The three alleles with the highest AF% values in the dataset associated with the MHP phenotype were PAH0562 [c.1208C>T (p.Ala403Val)] with a frequency of 2.03%, PAH0342 [c.898G >T (p.Ala300Ser)] with a frequency of 1.57%, and PAH0668 [c.158G>A (p.Arg53His)] with a frequency of 1.02%.

Additionally, the low-frequency near-splice region variants PAH0683, PAH0467, PAH0682, and PAH0968 were associated with the cPKU phenotype. The AF% values ranged from 0.002 to 0.075, and the distance of mutations from the CSS ranged from 10 bp to 19 bp. However, within this distance, variants associated with the mPKU and MHP phenotypes were also observed (PAH2318 and PAH0551, respectively). The c.912G>A allele (PAH0352) was the only *PAH* variant associated with mPKU in the synonymous category. The synonymous nucleotide substitution was localized in the last triplet of exon 8, encoding glutamine (p.Gln304=). Consistent with the PAH0540 variant, the low allele frequency of this variant (AF = 0.059%) likely explains why it has not been previously investigated.

## 3. Discussion

In the current study, we conducted an analytical review of the Phenylalanine Hydroxylase Gene Locus-Specific Database (PAHvdb). In the past 4 years (2022–2026), PAHvdb expanded by 169.26%, increasing from 1285 to 3460 variants, highlighting the increasing reliance on this database and underscoring the need for a systematic review of newly added variants. Despite this, to our knowledge, no other resource provides such a comprehensive representation of *PAH* variant data, including databases such as LOVD 3 (https://databases.lovd.nl/shared/genes/PAH (accessed on 20 April 2026)), ClinVar (https://www.ncbi.nlm.nih.gov/clinvar (accessed on 20 April 2026)), and GeneBe (https://genebe.net/gene/hg38/PAH (accessed on 20 April 2026)). A distinctive feature of the BIOPKU and PAHvdb databases is their ability to collect and dynamically calculate important metrics such as EA%, AF%, and APV. Thus, PAHvdb is currently the most complete and up-to-date source of information on the diversity of pathogenic mutations associated with PKU.

The most important results of our study are the determination of the analytical and phenotypic structure of the PAHvdb, as well as the demonstration of the degree of deficiency (gaps) in the annotation data of most *PAH* variants. Of the 3409 variants studied, 75.68% (*n* = 2580) could not be assigned a phenotype due to the fact that APVs were available for only 24.31% (*n* = 829) of the variants. Analysis of cPKU phenotypes in the splice donor–acceptor and splice-region categories indicated that variants associated with splicing defects represented approximately 4.37% of the variants in the database. EA% data, which provide the most direct biochemical evidence for genotype–phenotype inference, were available for only 3.84% (*n* = 131) of the variants. Of these, 97 variants had both APV and EA% values. Despite these limited data, the positive Spearman’s rank correlation coefficient (ρ) between EA% and APV (ρ = 0.796, *p* = 2.03 × 10^−22^) was consistent with the known relationship between residual PAH enzymatic activity and the severity of the metabolic phenotype [3].

Although the relative proportions of all mutation types in our results align with previous findings [19,20], the proportion of the indel type appears underestimated due to database classification conventions. Apparently, many variants were assigned to the deletion type that actually belong to the indel type. For example, examination of the values in the “cDNA Aberration (NM_000277.3)” shows that numerous single-nucleotide events are categorized strictly as deletions rather than indels. From this perspective, this category would represent the second most frequent variant type in the *PAH* gene following substitutions. Conversely, if insertions and deletions are isolated into mutually exclusive categories by removing the overlapping ‘indel’ designation entirely, the deletion type ranks second in abundance, reconciling our data with previously published literature. Clarification of this issue depends on the accepted classification of mutation types used in the database.

Currently, in PAHvdb, variants belonging to the near-splice region category are quantitatively comparable to missense mutations. From the very beginning, both pathogenic and non-coding mutations of the *PAH* gene have been registered in the database [21]. In a 2014 review study [16], most *PAH* variants were located in exons rather than introns. However, their relative proportions are now approximately equal (51.63% in exons, 48.37% in introns). Apparently, the use of primer panels in NGS that broadly cover adjacent intronic regions, together with their low selective pressure, led to the observed multitude of variants in the near-splice category.

It has been repeatedly mentioned that the majority of PKU cases are caused by missense mutations [3]. The results shown in Figure 4 are consistent with this finding. Interestingly, the number of missense mutations in known phenotypes (*n* = 284) is less than the sum of *PAH* variants from other categories associated only with the cPKU phenotype (*n* = 447; Figure 2, Table S3). This indicates that the prominent role of missense mutations in the number of PKU cases is due to the frequency of certain alleles and not to the degree of diversity of missense mutations in the *PAH* gene.

The *PAH* allele frequency spectrum of the dataset displayed pronounced asymmetry, characterized by a small number of high AF% alleles and a ‘long tail’ of rare variants. Notably, over 50% of the variants identified across all *PAH* exons possessed low AF values, which likely accounts for their lack of prior functional characterization or established phenotypic associations (Figure 3, Table S4). This observation demonstrates a significant gap in annotation data for most variants deposited in the PAHvdb. The reasons for the lack of annotation data for variants with low AF% are both practical and epidemiological.

From a practical point of view, the difficulty of reliably annotating variants lies in the fact that semi-empirical APVs are based on statistical data on patients’ genotypes and clinical phenotypes, as well as on residual enzymatic activity (EA%). APVs are calculated based on the frequency of the metabolic phenotype (i.e., cPKU, mPKU, or MHP) for genotypes that are in a functionally hemizygous state (in combination with an inactive null allele) [3]. As the number of observed patients with an identical *PAH* allele increases, and depending on their metabolic phenotype, the APV for this variant in the database is established and dynamically adjusted. However, the majority of patients (73%) are compound heterozygotes, whose PKU severity is mitigated by interallelic complementation [20,22]. Also, the phenotype can be influenced by diet or pharmacological treatment [2]. Determination of the clinical phenotype requires dynamic measurement of blood Phe levels before treatment [8]. However, the difficulty in obtaining these data stems from the need to start a diet in the first days of newborns’ lives [2]. The most reliable approach to determining EA% is to analyze the biochemical properties of the recombinant protein corresponding to a specific *PAH* variant expressed in a human cell model [23]. However, this approach is technically complex and has been performed only for a small number of variants [24]. Previously, an attempt was made to predict EA% and other *PAH* characteristics using an in silico approach based on allele genotypes [25]. However, the approach was limited to missense variants, of which 37% were hypothetically resolved.

From an epidemiological perspective, the frequency and prevalence of *PAH* alleles vary by ethnicity, geographic region, and the genetic and migratory histories of specific populations [10,20]. For example, the total pool of *PAH* mutant alleles in the Han population consisted of a limited number of frequently reported mutations and a very large number of rare mutations [26]. In an analysis of the epidemiology of PKU, it was determined that about 55% of the different *PAH* genotypes were found in individual patients [20]. This shows that the variants that form the proportions of alleles of unknown phenotype in the exons of the *PAH* gene are low-frequency mutations circulating in populations (AF% < 0.04). This means they are found only in individual patients or small families, which limits the statistical power to reliably detect APV. It is also worth noting that some proportion of alleles with low AF% may constitute de novo mutations [26,27]. Unfortunately, their number is uncertain, since such alleles are determined by comparison with patients’ parental alleles, a practice not generally accepted [21,26]. Ultimately, clinicians must rely solely on the baseline Phe level, which delays optimization of the treatment strategy and limits the ability to predict response to BH_4_.

Continued international collaboration is critical given the long tail of rare *PAH* variants and the large proportion of variants lacking phenotype information. Clinics, diagnostic laboratories, and research centers are encouraged to submit standardized baseline (pre-treatment) blood phenylalanine (Phe) concentrations, reports of novel *PAH* variants, and relevant clinical metadata to publicly accessible databases, where possible. Standardized reporting would enable genotype–phenotype annotation, enhance the accuracy of allelic phenotype value (APV) estimation, and facilitate more reliable prediction of phenotype from genotype for people with phenylketonuria.

The National PKU Alliance’s recent acquisition of BIOPKU/PAHvdb is a major step toward the long-term sustainability and continued development of this resource. Centralized stewardship may enable standardized data curation, harmonization of genotype–phenotype annotations, and better interoperability with other genomic and clinical databases. Future integration with clinical data sources such as electronic health records and patient registries will facilitate more extensive phenotype annotation and help to reduce the large proportion of variants for which phenotype information is presently lacking. Such improvements would increase the utility of PAHvdb for clinical decision-making and research, but such features have not yet been implemented.

This study has several limitations. First, all downstream analyses are inherently dependent on the accuracy, completeness, and curation standards of the PAHvdb and BIOPKU repositories; thus, the phenotypic classifications presented herein reflect only the records archived in these specific systems. Furthermore, the incomplete data in the registry—specifically the exclusion of the 51 variants lacking annotated records—may have introduced bias in the distribution of *PAH* variants with allele frequencies >1% (Figure 4). Second, the allele frequencies (AF%) utilized represent global metrics dynamically computed within the PAHvdb rather than true, unselected population-level frequencies. These values are subject to geographic ascertainment bias based on the specific ancestral composition of the BIOPKU registry cohort. Consequently, our findings should be interpreted as a descriptive characterization of the current *PAH* database landscape rather than an independent, clinically validated classification of *PAH* variant pathogenicity.

## 4. Materials and Methods

### 4.1. Data Source and Database Access

The study was conducted using descriptive statistical methods based on 3409 *PAH* gene variants manually downloaded from the locus-specific PAHvdb database, which is linked to the BIOPKU genotype–phenotype resource and available from www.biopku.org/home/pah.asp (accessed on 20 April 2026). The reference accession number for the *PAH* gene included Ensembl ID ENSG00000171759, and NCBI RefSeq accession numbers NC_000012.12 (genomic) and NM_000277.3 (mRNA). In addition to standard variant data, PAHvdb accumulates detailed information on affected protein domains, in vitro enzymatic activity, cofactor-binding regions (CBRs), and substrate-binding regions (SDBRs). The database also includes allelic phenotype values (APVs), allele frequencies, ClinVar annotations, REVEL scoring, and bibliographic references.

A local working copy of PAHvdb was generated by manually copying all available *PAH* variant records into a structured tabular format (collection date: 30 April 2026). All data were stored in the original PAHvdb annotation format and are available in Supplementary Materials Files S1 and S2. The initial PAHvdb list contained data records for a total of 3460 variants. Records without annotations to unambiguously identify and classify the reported variant were excluded, as they could not be included reliably in analyses of variant type, genomic distribution, or genotype–phenotype associations. Therefore, 51 records (Table S1) were removed, and the final dataset consisted of 3409 variants for analysis.

The following annotation fields were extracted and used in the statistical analysis: “PAH Identifier”, “Protein Variant”, “cDNA Aberration (NM_000277.3)”, “Variant Type”, “Coding Effect”, “Gene Region”, “Protein Domain”, “Enzyme Activity (%)”, “Allelic Phenotype Value (APV)”, and “Allele Frequency (%)”. The field names and their values remained consistent with the original PAHvdb nomenclature to avoid introducing systematic classification errors during data processing.

Values in the fields “Allelic Phenotype Values (APVs)” and “Allele frequency (AF%)” were calculated dynamically based on anonymized genotype and corresponding phenotype data of 33914 HPA patients in the BIOPKU database (www.biopku.org) [3]. Values classified as “Allelic Phenotype Value (APV (Weak))” were excluded from the analysis, as they correspond to *PAH* variants with very low frequency and are statistically unreliable.

### 4.2. Data Preprocessing

The entire dataset was checked for duplicate values in the “PAH Identifier” field. The entire dataset contains missing values (“NaN”, “Unknown”, and “−11”) in the “Allelic” field. “Allelic Phenotype Values (APVs)” were labeled as “Unknown”. Phenotype assignment for each *PAH* variant was performed similarly to the previously adopted APVs [3], as follows: cPKU (APV = 0–2.7), mPKU (APV = 2.8–6.6), and MHP (APV = 6.7–10). The assigned phenotype values were automatically recorded in the new field “Assigned Phenotype” depending on the APVs. Variants with unknown APV were assigned the value “Unknown” in the “Assigned Phenotype” field. This approach was used to describe the phenotypic structure of PAHvdb and was not intended to independently determine the clinical pathogenicity of variants for which there is insufficient evidence of a genotype–phenotype association.

### 4.3. Statistical Analysis

All analyses were performed in the Jupyter Notebook v7.5.5 environment, with Python v3.14.4 (python.org). Data processing and summary statistics were performed using Pandas v3.0.2 (pandas.pydata.org) and NumPy v2.0.0 (numpy.org). Statistical analysis was performed using SciPy v1.17.1 (scipy.org), and plots were generated using Matplotlib v3.10.8 (matplotlib.org).

Spearman’s rank correlation coefficient (ρ) was used to assess the correlation between EA% and APV, as APV is an ordinal, semiempirical metric with discrete threshold values. To assess the influence of the two variants with reported enzymatic activity (EA%) values exceeding 100% (111% and 123%), a sensitivity analysis was performed by repeating the correlation analysis twice: first using the complete dataset and then after excluding these two observations. Spearman’s rank correlation coefficient (ρ) was calculated for both analyses, and the results were compared to evaluate the robustness of the observed association. Statistical significance was evaluated as a two-sided *p*-value < 0.05.

## Data Availability

The PAHvdb-derived dataset used in this study is provided in Table S2, and the analysis scripts are provided in Supplementary Material S2.

## Supplementary Materials

The following supporting information can be downloaded at: https://www.mdpi.com/article/10.3390/ijms27156772/s1.
