# Supplementary material for: Analysis of PAH Genetic Variation and Phenotypic Diversity in the PAHvdb
Source: Int J Mol Sci. 2026 Jul 29;27(15):6772. doi: 10.3390/ijms27156772 (PMC13467348; doi:10.3390/ijms27156772)
Supplement: Supplementary file 1 [file ijms-27-06772-s001.zip › Supplementary Material File S2.html]

PAH\_pipeline\_v0.3


In [1]:

```
# Importing modules
import os
import pandas as pd
import matplotlib.pyplot as plt

# Display all columns of dataframe
pd.options.display.max_columns = None
```

### 0. Turn the raw table over and prepare it for analysis.¶

In [2]:

```
# Importning raw table
df = pd.read_csv("db_raw/PAHvdb_collected_checked_v2_raw.tsv", sep='\t', header=None, dtype = str)
df = df.T
df.columns = df.iloc[0]
df = df[1:]
raw_table = df
#raw_table

# Preparing table for analysis
# Extract needed columns
PAHvdb_table = raw_table.drop(raw_table.columns[[0, 1, 2, 3, 6, 8, 9, 10, 11, 16, 17, 18, 19, 40, 42]], axis = 1).copy()
# to remove table from memory
del raw_table

PAHvdb_table
```

Out[2]:

|  | PAH Identifier | Protein Variant | cDNA Aberration (NM\_000277.3) | Variant Type | Coding Effect | Gene Region | Protein Domain | CADD | 3D Structural Prediction | SPiP | SplicAI | BIOPKU Phenotype | Enzyme Activity (%) | Allelic Phenotype Value (APV) | Allelic Phenotype Value (APV (Weak)) | Allele Frequency (%) | REVEL | GnomAD | BIOPKU ACMG/AMP/ACGS Interpretation | BIOPKU ACMG/AMP/ACGS scores | ClinVar ACMG Interpretation | ClinVar ACMG Scores | ClinVarID | FRANKLIN | FRANKLIN scores | VARSOME | VARSOME scores | References |
| --- | --- | --- | --- | --- | --- | --- | --- | --- | --- | --- | --- | --- | --- | --- | --- | --- | --- | --- | --- | --- | --- | --- | --- | --- | --- | --- | --- | --- |
| 1 | PAH0882 | p.(=) / -626G>A | c.-626G>A | Substitution | Unknown | 5'UTR | Not Known | NaN | NaN | NaN | NaN | NaN | NaN | -11 | NaN | NaN | NaN | NaN | NaN | NaN | NaN | NaN | NaN | NaN | NaN | NaN | NaN | Yu, W., J. He, X. Yang, H. Zou, J. Gui, R. Wan... |
| 2 | PAH0883 | p.(=) / -480\_-478del | c.-480\_-478del | Deletion | Unknown | 5'UTR | Not Known | NaN | NaN | NaN | NaN | NaN | NaN | -11 | NaN | NaN | NaN | NaN | NaN | NaN | NaN | NaN | NaN | NaN | NaN | NaN | NaN | Yu, W., J. He, X. Yang, H. Zou, J. Gui, R. Wan... |
| 3 | PAH1798 | p.(=) / -95-385\_-95-382del | c.-95-385\_-95-382del | Deletion | Unknown | 5'UTR | Not Known | NaN | NaN | NaN | NaN | NaN | NaN | -11 | NaN | NaN | NaN | NaN | NaN | NaN | NaN | NaN | NaN | NaN | NaN | NaN | NaN | ClinVar |
| 4 | PAH1799 | p.(=) / -460del | c.-460del | Deletion | Unknown | 5'UTR | Not Known | NaN | NaN | NaN | NaN | NaN | NaN | -11 | NaN | NaN | NaN | NaN | NaN | NaN | NaN | NaN | NaN | NaN | NaN | NaN | NaN | ClinVar |
| 5 | PAH1795 | p.(=) / -95-4071\_-95-313del | c.-95-4071\_-95-313del | Deletion | Unknown | 5'UTR | Not Known | NaN | NaN | NaN | NaN | NaN | NaN | -11 | NaN | NaN | NaN | NaN | NaN | NaN | NaN | NaN | NaN | NaN | NaN | NaN | NaN | ClinVar |
| ... | ... | ... | ... | ... | ... | ... | ... | ... | ... | ... | ... | ... | ... | ... | ... | ... | ... | ... | ... | ... | ... | ... | ... | ... | ... | ... | ... | ... |
| 3405 | PAH0937 | p.(Glu78Val) | c.233A>T | Substitution | Missense | E3 | regulatory | 31 | LP | No effect on splicing | No impact | NaN | NaN | -11 | NaN | NaN | 0.824 | 1/ 628758 | PM2+PM5+PP3\_mod.+PP4\_mod.+PM1 | P | VCEP: VUS (PP4\_Moderate, PM2, PP3) | VUS | 872831 | PM1-Mod, PP2-Sup, PM2-Mod, PM5-Mod, PP3-Sup | LP | PM1-ST, PM2-Sup, PM5-Mod, PP3-ST | P | Li, N., H. Jia, Z. Liu, J. Tao, S. Chen, X. Li... |
| 3406 | PAH1566 | p.(Glu78Asp) | c.234A>T | Substitution | Missense | E3 | regulatory | 14.17 | NaN | No effect on splicing | No impact | NaN | NaN | -11 | NaN | NaN | 0.676 | 0 | PM2+PM5+PM1 | LP | NaN | NaN | 1443943 | PM1-Mod, PP2-Sup, PM2-Mod, PM5-Mod, PP3-Sup | LP | PM1-ST, PM2-Sup, PM5-Mod; PP3-Mod | LP | ClinVar |
| 3407 | PAH0610 | p.(=) / IVS12+6T>A | c.1315+6T>A | Substitution | Splice Region | I12 | Not Known | NaN | NaN | NaN | NaN | NaN | NaN | 5.3 | NaN | 0.137 | NaN | NaN | NaN | NaN | NaN | NaN | NaN | NaN | NaN | NaN | NaN | Song, F., Y. J. Qu, T. Zhang, Y. W. Jin, H. Wa... |
| 3408 | PAH3442 | p.(=) | c.\*34A>T | Substitution | Missense | 3'UTR | Not Known | 14.17 | NaN | No effect on splicing | No impact | NaN | NaN | -11 | NaN | NaN | 0.676 | 0 | PM2+PM5+PM1 | LP | NaN | NaN | 1443943 | PM1-Mod, PP2-Sup, PM2-Mod, PM5-Mod, PP3-Sup | LP | PM1-ST, PM2-Sup, PM5-Mod; PP3-Mod | LP | gnomAD 4.0 (https://gnomad.broadinstitute.org/) |
| 3409 | PAH3449 | p.(=) | c.\*43T>C | Substitution | Missense | 3'UTR | Not Known | 25.7 | LP | No effect on splicing | No impact | cPKU | NaN | -11 | NaN | NaN | 0.97 | 142/ 1613302 | PM2+PP3\_mod.+PS3+PP1+PP4\_mod.+PM3\_verystrong+PM1 | P | VCEP: P (PP4\_Moderate, PS3, PM3, PP1, PP3) | P | 608 | PS3-ST; PM1-Mod; PP2-Sup; PM2-Mod; PP3-Mod; PP... | P | PS3-ST; PM1-ST; PM2-Sup; PP3-Mod; PP5-VST | P | gnomAD 4.0 (https://gnomad.broadinstitute.org/) |

3409 rows × 28 columns

In [3]:

```
# find duplicate values ​​in the "PAH Identifier" column
#duplicates = PAHvdb_table["PAH Identifier"][PAHvdb_table["PAH Identifier"].duplicated()].unique()
#print(duplicates)

# 0.1 Reassign APV to a new phenotype
# Adding a new empty column
PAHvdb_table["Assigned phenotype"] = ''
#PAHvdb_table

for i in range(len(PAHvdb_table)):
    value = PAHvdb_table.iloc[i, 13]

    # "Not_Known" processing
    if pd.isna(value) or str(value) == "-11":
        PAHvdb_table.iloc[i, 28] = "Unknown"

    else:
        try:
            v = float(value)

            if 0 <= v <= 2.7:
                PAHvdb_table.iloc[i, 28] = "cPKU"
            elif 2.8 <= v <= 6.6:
                PAHvdb_table.iloc[i, 28] = "mPKU"
            elif 6.7 <= v <= 10:
                PAHvdb_table.iloc[i, 28] = "MHP"
            else:
                print("вне диапазонов") # Signal
                PAHvdb_table.iloc[i, 28] = np.nan  # if outside the ranges

        except:
            print("Что-то не так") # Signal

PAHvdb_table
```

Out[3]:

|  | PAH Identifier | Protein Variant | cDNA Aberration (NM\_000277.3) | Variant Type | Coding Effect | Gene Region | Protein Domain | CADD | 3D Structural Prediction | SPiP | SplicAI | BIOPKU Phenotype | Enzyme Activity (%) | Allelic Phenotype Value (APV) | Allelic Phenotype Value (APV (Weak)) | Allele Frequency (%) | REVEL | GnomAD | BIOPKU ACMG/AMP/ACGS Interpretation | BIOPKU ACMG/AMP/ACGS scores | ClinVar ACMG Interpretation | ClinVar ACMG Scores | ClinVarID | FRANKLIN | FRANKLIN scores | VARSOME | VARSOME scores | References | Assigned phenotype |
| --- | --- | --- | --- | --- | --- | --- | --- | --- | --- | --- | --- | --- | --- | --- | --- | --- | --- | --- | --- | --- | --- | --- | --- | --- | --- | --- | --- | --- | --- |
| 1 | PAH0882 | p.(=) / -626G>A | c.-626G>A | Substitution | Unknown | 5'UTR | Not Known | NaN | NaN | NaN | NaN | NaN | NaN | -11 | NaN | NaN | NaN | NaN | NaN | NaN | NaN | NaN | NaN | NaN | NaN | NaN | NaN | Yu, W., J. He, X. Yang, H. Zou, J. Gui, R. Wan... | Unknown |
| 2 | PAH0883 | p.(=) / -480\_-478del | c.-480\_-478del | Deletion | Unknown | 5'UTR | Not Known | NaN | NaN | NaN | NaN | NaN | NaN | -11 | NaN | NaN | NaN | NaN | NaN | NaN | NaN | NaN | NaN | NaN | NaN | NaN | NaN | Yu, W., J. He, X. Yang, H. Zou, J. Gui, R. Wan... | Unknown |
| 3 | PAH1798 | p.(=) / -95-385\_-95-382del | c.-95-385\_-95-382del | Deletion | Unknown | 5'UTR | Not Known | NaN | NaN | NaN | NaN | NaN | NaN | -11 | NaN | NaN | NaN | NaN | NaN | NaN | NaN | NaN | NaN | NaN | NaN | NaN | NaN | ClinVar | Unknown |
| 4 | PAH1799 | p.(=) / -460del | c.-460del | Deletion | Unknown | 5'UTR | Not Known | NaN | NaN | NaN | NaN | NaN | NaN | -11 | NaN | NaN | NaN | NaN | NaN | NaN | NaN | NaN | NaN | NaN | NaN | NaN | NaN | ClinVar | Unknown |
| 5 | PAH1795 | p.(=) / -95-4071\_-95-313del | c.-95-4071\_-95-313del | Deletion | Unknown | 5'UTR | Not Known | NaN | NaN | NaN | NaN | NaN | NaN | -11 | NaN | NaN | NaN | NaN | NaN | NaN | NaN | NaN | NaN | NaN | NaN | NaN | NaN | ClinVar | Unknown |
| ... | ... | ... | ... | ... | ... | ... | ... | ... | ... | ... | ... | ... | ... | ... | ... | ... | ... | ... | ... | ... | ... | ... | ... | ... | ... | ... | ... | ... | ... |
| 3405 | PAH0937 | p.(Glu78Val) | c.233A>T | Substitution | Missense | E3 | regulatory | 31 | LP | No effect on splicing | No impact | NaN | NaN | -11 | NaN | NaN | 0.824 | 1/ 628758 | PM2+PM5+PP3\_mod.+PP4\_mod.+PM1 | P | VCEP: VUS (PP4\_Moderate, PM2, PP3) | VUS | 872831 | PM1-Mod, PP2-Sup, PM2-Mod, PM5-Mod, PP3-Sup | LP | PM1-ST, PM2-Sup, PM5-Mod, PP3-ST | P | Li, N., H. Jia, Z. Liu, J. Tao, S. Chen, X. Li... | Unknown |
| 3406 | PAH1566 | p.(Glu78Asp) | c.234A>T | Substitution | Missense | E3 | regulatory | 14.17 | NaN | No effect on splicing | No impact | NaN | NaN | -11 | NaN | NaN | 0.676 | 0 | PM2+PM5+PM1 | LP | NaN | NaN | 1443943 | PM1-Mod, PP2-Sup, PM2-Mod, PM5-Mod, PP3-Sup | LP | PM1-ST, PM2-Sup, PM5-Mod; PP3-Mod | LP | ClinVar | Unknown |
| 3407 | PAH0610 | p.(=) / IVS12+6T>A | c.1315+6T>A | Substitution | Splice Region | I12 | Not Known | NaN | NaN | NaN | NaN | NaN | NaN | 5.3 | NaN | 0.137 | NaN | NaN | NaN | NaN | NaN | NaN | NaN | NaN | NaN | NaN | NaN | Song, F., Y. J. Qu, T. Zhang, Y. W. Jin, H. Wa... | mPKU |
| 3408 | PAH3442 | p.(=) | c.\*34A>T | Substitution | Missense | 3'UTR | Not Known | 14.17 | NaN | No effect on splicing | No impact | NaN | NaN | -11 | NaN | NaN | 0.676 | 0 | PM2+PM5+PM1 | LP | NaN | NaN | 1443943 | PM1-Mod, PP2-Sup, PM2-Mod, PM5-Mod, PP3-Sup | LP | PM1-ST, PM2-Sup, PM5-Mod; PP3-Mod | LP | gnomAD 4.0 (https://gnomad.broadinstitute.org/) | Unknown |
| 3409 | PAH3449 | p.(=) | c.\*43T>C | Substitution | Missense | 3'UTR | Not Known | 25.7 | LP | No effect on splicing | No impact | cPKU | NaN | -11 | NaN | NaN | 0.97 | 142/ 1613302 | PM2+PP3\_mod.+PS3+PP1+PP4\_mod.+PM3\_verystrong+PM1 | P | VCEP: P (PP4\_Moderate, PS3, PM3, PP1, PP3) | P | 608 | PS3-ST; PM1-Mod; PP2-Sup; PM2-Mod; PP3-Mod; PP... | P | PS3-ST; PM1-ST; PM2-Sup; PP3-Mod; PP5-VST | P | gnomAD 4.0 (https://gnomad.broadinstitute.org/) | Unknown |

3409 rows × 29 columns

In [4]:

```
# 0.2 Save the working database to a file
PAHvdb_table.to_csv('results_3/PAHvdb_table_assigned_phenotypes.tsv', sep='\t', index=False)
```

In [11]:

```
# Prints index and name line-by-line
for i, col in enumerate(PAHvdb_table.columns):
    print(f"Index: {i}, Name: {col}")
```

```
Index: 0, Name: PAH Identifier
Index: 1, Name: Protein Variant
Index: 2, Name: cDNA Aberration (NM_000277.3)
Index: 3, Name: Variant Type
Index: 4, Name: Coding Effect
Index: 5, Name: Gene Region
Index: 6, Name: Protein Domain
Index: 7, Name: CADD
Index: 8, Name: 3D Structural Prediction
Index: 9, Name: SPiP
Index: 10, Name: SplicAI
Index: 11, Name: BIOPKU Phenotype
Index: 12, Name: Enzyme Activity (%)
Index: 13, Name: Allelic Phenotype Value (APV)
Index: 14, Name: Allelic Phenotype Value (APV (Weak))
Index: 15, Name: Allele Frequency (%)
Index: 16, Name: REVEL
Index: 17, Name: GnomAD
Index: 18, Name: BIOPKU ACMG/AMP/ACGS Interpretation
Index: 19, Name: BIOPKU ACMG/AMP/ACGS scores
Index: 20, Name: ClinVar ACMG Interpretation
Index: 21, Name: ClinVar ACMG Scores
Index: 22, Name: ClinVarID
Index: 23, Name: FRANKLIN
Index: 24, Name: FRANKLIN scores
Index: 25, Name: VARSOME
Index: 26, Name: VARSOME scores
Index: 27, Name: References
Index: 28, Name: Assigned phenotype
```

### 1. Variant types and their phenotypes. Statistics¶

In [15]:

```
# 
variant_type_list = PAHvdb_table.iloc[:, 3].tolist()
variant_type_set = set(variant_type_list)

for variant_type in variant_type_set:
    count = 0
    Unknown_value = 0
    cPKU_value = 0
    mPKU_value = 0
    MHP_value = 0
    for variant, phenotype in zip(PAHvdb_table.iloc[:, 3], PAHvdb_table.iloc[:, 28]):
        if variant_type == variant:
            count += 1
            if phenotype == "Unknown":
                Unknown_value += 1
            elif phenotype == "cPKU":
                cPKU_value += 1
            elif phenotype == "mPKU":
                mPKU_value += 1
            elif phenotype == "MHP":
                MHP_value += 1
    print("Number of variant", variant_type, count, sep = "  ")
    print("Unknown_value", Unknown_value, sep = " = ")
    print("cPKU_value", cPKU_value, sep = " = ")
    print("mPKU_value", mPKU_value, sep = " = ")
    print("MHP_value", MHP_value, sep = " = ")
```

```
Number of variant  Substitution  2969
Unknown_value = 2449
cPKU_value = 374
mPKU_value = 72
MHP_value = 74
Number of variant  Insertion  22
Unknown_value = 3
cPKU_value = 19
mPKU_value = 0
MHP_value = 0
Number of variant  Duplication  61
Unknown_value = 21
cPKU_value = 40
mPKU_value = 0
MHP_value = 0
Number of variant  Deletion  294
Unknown_value = 87
cPKU_value = 205
mPKU_value = 2
MHP_value = 0
Number of variant  Indel  28
Unknown_value = 15
cPKU_value = 12
mPKU_value = 0
MHP_value = 1
Number of variant  Deletion - Large  35
Unknown_value = 5
cPKU_value = 30
mPKU_value = 0
MHP_value = 0
```

### 1.1 Graph: Types of variants and their phenotypes¶

# 1. Results of counting the number of variant types
variant\_types = ["Substitution", "Deletion", "Duplication", "Deletion-Large", "Indel", "Insertion"]
variant\_types\_counts = [2969, 294, 61, 35, 28, 22]
variant\_types\_percentages = [87.1, 8.6, 1.8, 1.0, 0.8, 0.6]
# Sum = 3409

In [1]:

```
import numpy as np
import matplotlib.pyplot as plt

from matplotlib.patches import Patch


# Data
variant_types = ["Substitution", "Deletion", "Duplication", "Deletion-Large", "Indel", "Insertion"]
variant_types_counts = [2969, 294, 61, 35, 28, 22]
variant_types_percentages = [87.09, 8.62, 1.78, 1.02, 0.82, 0.64]

# 4 segments within each column
variant_types_percentages_phenotypes = [
    [82.5, 12.6, 2.4, 2.5], # Substitution
    [29.6, 69.7, 0.7, 0], # Deletion
    [34.4, 65.6, 0, 0], # Duplication
    [14.3, 85.7, 0, 0], # Deletion-Large
    [53.6, 42.9, 0, 3.6], # Indel
    [13.6, 86.4, 0, 0] # Insertion
]

segment_colors = ["#808080", "#C44E52", "#87CEEB", "#55A868"] # Unknown, cPKU, mPKU, MHP

fig, ax = plt.subplots()

x = np.arange(len(variant_types))
width = 0.5

# stacked bars
for i in range(4):
    bottom = np.zeros(len(variant_types))

    for j in range(i):
        bottom += np.array(variant_types_counts) * np.array([p[j] for p in variant_types_percentages_phenotypes]) / 100

    values = np.array(variant_types_counts) * np.array([p[i] for p in variant_types_percentages_phenotypes]) / 100

    ax.bar(
        x,
        values,
        width,
        bottom=bottom,
        color=segment_colors[i],
        edgecolor='black',
        linewidth=0.8
    )

# column captions
for i in range(len(variant_types)):
    ax.text(
        i,
        variant_types_counts[i] + max(variant_types_counts) * 0.02,
        f"{variant_types_percentages[i]}%\n{variant_types_counts[i]}",
        ha='center',
        va='bottom',
        fontsize=8 #fontweight='bold'
    )

# scale
ax.set_yscale('linear')

ax.get_yaxis().set_major_formatter(plt.ScalarFormatter())

ax.tick_params(axis='y', labelsize=8)

ax.set_xticks(x)
ax.set_xticklabels(variant_types, fontsize=8)

ax.set_xlabel("Variant type")
ax.set_ylabel("Number of variants")

ax.spines['top'].set_visible(False)
ax.spines['right'].set_visible(False)

ax.yaxis.grid(True, which='both', linestyle='', alpha=0.7)

# Legend
legend_elements = [
    Patch(facecolor=segment_colors[0], edgecolor='black', label='Unknown'),
    Patch(facecolor=segment_colors[1], edgecolor='black', label='cPKU'),
    Patch(facecolor=segment_colors[2], edgecolor='black', label='mPKU'),
    Patch(facecolor=segment_colors[3], edgecolor='black', label='MHP')
]

ax.legend(handles=legend_elements, loc='upper right', frameon=True)


plt.tight_layout()
plt.savefig("visualization_results/1_variant_types_distribution.png", dpi=300, bbox_inches='tight')
plt.show()
```

### 2. Mutation effects and their phenotypes. Statistics¶

In [102]:

```
#
# Пexamine the variety of values ​​in the "Coding Effect" column
Coding_effect_list = PAHvdb_table.iloc[:, 4].tolist()
Coding_effect_set = set(Coding_effect_list)

for Coding_effect in Coding_effect_set:
    count = 0
    Unknown_value = 0
    cPKU_value = 0
    mPKU_value = 0
    MHP_value = 0
    for effect, phenotype in zip(PAHvdb_table.iloc[:, 4], PAHvdb_table.iloc[:, 28]):
        if Coding_effect == effect:
            count += 1
            if phenotype == "Unknown":
                Unknown_value += 1
            elif phenotype == "cPKU":
                cPKU_value += 1
            elif phenotype == "mPKU":
                mPKU_value += 1
            elif phenotype == "MHP":
                MHP_value += 1
    print("Number of variant", Coding_effect, count, sep = "  ")
    print("Unknown_value", Unknown_value, sep = " = ")
    print("cPKU_value", cPKU_value, sep = " = ")
    print("mPKU_value", mPKU_value, sep = " = ")
    print("MHP_value", MHP_value, sep = " = ")
```

```
Number of variant  Unknown  94
Unknown_value = 94
cPKU_value = 0
mPKU_value = 0
MHP_value = 0
Number of variant  Nonsense  102
Unknown_value = 2
cPKU_value = 100
mPKU_value = 0
MHP_value = 0
Number of variant  Deep Intronic  83
Unknown_value = 76
cPKU_value = 4
mPKU_value = 2
MHP_value = 1
Number of variant  Missense  1104
Unknown_value = 820
cPKU_value = 147
mPKU_value = 64
MHP_value = 73
Number of variant  Synonimous  298
Unknown_value = 296
cPKU_value = 1
mPKU_value = 1
MHP_value = 0
Number of variant  Near-splice Region  1167
Unknown_value = 1158
cPKU_value = 7
mPKU_value = 1
MHP_value = 1
Number of variant  In-frame  35
Unknown_value = 13
cPKU_value = 21
mPKU_value = 1
MHP_value = 0
Number of variant  Initiation codon  7
Unknown_value = 0
cPKU_value = 5
mPKU_value = 2
MHP_value = 0
Number of variant  Splice Region  159
Unknown_value = 111
cPKU_value = 45
mPKU_value = 3
MHP_value = 0
Number of variant  Extension  4
Unknown_value = 1
cPKU_value = 3
mPKU_value = 0
MHP_value = 0
Number of variant  Frame shift  260
Unknown_value = 0
cPKU_value = 260
mPKU_value = 0
MHP_value = 0
Number of variant  Splice Donor-Aceptor  96
Unknown_value = 9
cPKU_value = 87
mPKU_value = 0
MHP_value = 0
```

### 2.1 Graph: Mutation effects and their phenotypes¶

# 2. Results of calculating the number of mutation effects
Coding\_effect = ["Near-splice Region", "Missense", "Synonimous", "Frame shift", "Splice Region", "Nonsense", "Splice Donor-Aceptor", "Unknown", "Deep Intronic", "In-frame", "Initiation codon", "Extension"]
Coding\_effect\_count = [1167, 1104, 298, 260, 159, 102, 96, 94, 83, 35, 7, 4]
Coding\_effect\_percentages = [34.25, 32.39, 8.74, 7.63, 4.66, 2.99, 2.82, 2.76, 2.44, 1.03, 0.21, 0.12]
# Sum = 3409

In [1]:

```
import numpy as np
import matplotlib.pyplot as plt

from matplotlib.patches import Patch


# Data
variant_types = [
    "Near-splice\nRegion",
    "Missense",
    "Synonymous",
    "Frame\nshift",
    "Splice\nRegion",
    "Nonsense",
    "Splice Donor-\nAcceptor",
    "Unknown",
    "Deep\nIntronic",
    "In-frame",
    "Initiation\ncodon",
    "Extension"
]

variant_types_counts = [1167, 1104, 298, 260, 159, 102, 96, 94, 83, 35, 7, 4]
variant_types_percentages = [34.25, 32.39, 8.74, 7.63, 4.66, 2.99, 2.82, 2.76, 2.44, 1.03, 0.21, 0.12]

# 4 segments within each column
variant_types_percentages_phenotypes = [
    [99.23, 0.6, 0.09, 0.09], # Near-splice Region
    [74.28, 13.32, 5.8, 6.61], # Missense
    [99.33, 0.34, 0.34, 0], # Synonimous
    [0, 100, 0, 0], # Frame shift
    [69.81, 28.3, 1.89, 0], # Splice Region
    [1.96, 98.04, 0, 0], # Nonsense
    [9.38, 90.62, 0, 0], # Splice Donor-Aceptor
    [100, 0, 0, 0], # Unknown
    [91.57, 4.82, 2.41, 1.2], # Deep Intronic
    [37.14, 60, 2.86, 0], # In-frame
    [0, 71.43, 28.57, 0], # Initiation codon
    [25, 75, 0, 0] # Extension
]

segment_colors = ["#808080", "#C44E52", "#87CEEB", "#55A868"] # Unknown, cPKU, mPKU, MHP

fig, ax = plt.subplots()

x = np.arange(len(variant_types))
width = 0.5

# stacked bars
for i in range(4):
    bottom = np.zeros(len(variant_types))

    for j in range(i):
        bottom += np.array(variant_types_counts) * np.array([p[j] for p in variant_types_percentages_phenotypes]) / 100

    values = np.array(variant_types_counts) * np.array([p[i] for p in variant_types_percentages_phenotypes]) / 100

    ax.bar(
        x,
        values,
        width,
        bottom=bottom,
        color=segment_colors[i],
        edgecolor='black',
        linewidth=0.8
    )

# column captions
for i in range(len(variant_types)):
    ax.text(
        i,
        variant_types_counts[i] + max(variant_types_counts) * 0.02,
        f"{variant_types_percentages[i]}%\n{variant_types_counts[i]}",
        ha='center',
        va='bottom',
        fontsize=8 #fontweight='bold'
    )

# scale
ax.set_yscale('linear')

ax.get_yaxis().set_major_formatter(plt.ScalarFormatter())

ax.tick_params(axis='y', labelsize=8)

ax.set_xticks(x)
ax.set_xticklabels(variant_types, fontsize=7)

ax.set_xlabel("Coding effect")
ax.set_ylabel("Number of variants")

ax.spines['top'].set_visible(False)
ax.spines['right'].set_visible(False)

ax.yaxis.grid(True, which='both', linestyle='', alpha=0.7)

# Legend
legend_elements = [
    Patch(facecolor=segment_colors[0], edgecolor='black', label='Unknown'),
    Patch(facecolor=segment_colors[1], edgecolor='black', label='cPKU'),
    Patch(facecolor=segment_colors[2], edgecolor='black', label='mPKU'),
    Patch(facecolor=segment_colors[3], edgecolor='black', label='MHP')
]

ax.legend(handles=legend_elements, loc='upper right', frameon=True)

plt.xticks(rotation=45)

plt.tight_layout()
plt.savefig("visualization_results/2_coding_effects_distribution.png", dpi=300, bbox_inches='tight')
plt.show()
```

### 3. Location of mutations in the gene and their phenotypes. Statistics¶

In [5]:

```
#
# View the variety of values ​​in the "Gene Region" column
Gene_region_list = PAHvdb_table.iloc[:, 5].tolist()
Gene_region_set = set(Gene_region_list)


for Gene_region in Gene_region_set:
    count = 0
    Unknown_value = 0
    cPKU_value = 0
    mPKU_value = 0
    MHP_value = 0
    for effect, phenotype in zip(PAHvdb_table.iloc[:, 5], PAHvdb_table.iloc[:, 28]):
        if Gene_region == effect:
            count += 1
            if phenotype == "Unknown":
                Unknown_value += 1
            elif phenotype == "cPKU":
                cPKU_value += 1
            elif phenotype == "mPKU":
                mPKU_value += 1
            elif phenotype == "MHP":
                MHP_value += 1
    print("Number of variant", Gene_region, count, sep = "  ")
    print("Unknown_value", Unknown_value, sep = " = ")
    print("cPKU_value", cPKU_value, sep = " = ")
    print("mPKU_value", mPKU_value, sep = " = ")
    print("MHP_value", MHP_value, sep = " = ")
```

```
Number of variant  E8  80
Unknown_value = 49
cPKU_value = 23
mPKU_value = 3
MHP_value = 5
Number of variant  I2  137
Unknown_value = 123
cPKU_value = 14
mPKU_value = 0
MHP_value = 0
Number of variant  I3  115
Unknown_value = 108
cPKU_value = 7
mPKU_value = 0
MHP_value = 0
Number of variant  I8  116
Unknown_value = 104
cPKU_value = 12
mPKU_value = 0
MHP_value = 0
Number of variant  E13  36
Unknown_value = 27
cPKU_value = 8
mPKU_value = 1
MHP_value = 0
Number of variant  5'UTR  83
Unknown_value = 81
cPKU_value = 2
mPKU_value = 0
MHP_value = 0
Number of variant  E9  86
Unknown_value = 51
cPKU_value = 20
mPKU_value = 8
MHP_value = 7
Number of variant  E12  146
Unknown_value = 97
cPKU_value = 35
mPKU_value = 9
MHP_value = 5
Number of variant  E11  186
Unknown_value = 104
cPKU_value = 63
mPKU_value = 9
MHP_value = 10
Number of variant  I11  148
Unknown_value = 132
cPKU_value = 15
mPKU_value = 0
MHP_value = 1
Number of variant  I9  120
Unknown_value = 107
cPKU_value = 13
mPKU_value = 0
MHP_value = 0
Number of variant  I12  123
Unknown_value = 110
cPKU_value = 12
mPKU_value = 1
MHP_value = 0
Number of variant  E2  121
Unknown_value = 83
cPKU_value = 34
mPKU_value = 0
MHP_value = 4
Number of variant  E7  202
Unknown_value = 117
cPKU_value = 72
mPKU_value = 7
MHP_value = 6
Number of variant  3'UTR  52
Unknown_value = 52
cPKU_value = 0
mPKU_value = 0
MHP_value = 0
Number of variant  I10  128
Unknown_value = 112
cPKU_value = 14
mPKU_value = 1
MHP_value = 1
Number of variant  I4  108
Unknown_value = 89
cPKU_value = 17
mPKU_value = 2
MHP_value = 0
Number of variant  E6  270
Unknown_value = 162
cPKU_value = 87
mPKU_value = 5
MHP_value = 16
Number of variant  I6  131
Unknown_value = 121
cPKU_value = 9
mPKU_value = 1
MHP_value = 0
Number of variant  E10  118
Unknown_value = 73
cPKU_value = 40
mPKU_value = 5
MHP_value = 0
Number of variant  I1  136
Unknown_value = 128
cPKU_value = 8
mPKU_value = 0
MHP_value = 0
Number of variant  I5  133
Unknown_value = 121
cPKU_value = 12
mPKU_value = 0
MHP_value = 0
Number of variant  E1  83
Unknown_value = 56
cPKU_value = 25
mPKU_value = 2
MHP_value = 0
Number of variant  E3  232
Unknown_value = 143
cPKU_value = 66
mPKU_value = 11
MHP_value = 12
Number of variant  E4  92
Unknown_value = 66
cPKU_value = 19
mPKU_value = 5
MHP_value = 2
Number of variant  I7  119
Unknown_value = 104
cPKU_value = 14
mPKU_value = 1
MHP_value = 0
Number of variant  E5  108
Unknown_value = 60
cPKU_value = 39
mPKU_value = 3
MHP_value = 6
```

### 3.1 Graph: Location of mutations in a gene and their phenotypes¶

In [35]:

```
import numpy as np
import matplotlib.pyplot as plt

from matplotlib.patches import Patch

# Data
variant_types = ["5'UTR", "E1", "I1", "E2", "I2", "E3", "I3", "E4", "I4",
               "E5", "I5", "E6", "I6", "E7", "I7", "E8", "I8", "E9", "I9",
               "E10", "I10", "E11", "I11", "E12", "I12", "E13", "3'UTR"]

variant_types_counts = [83, 83, 136, 121, 137, 232, 115, 92, 108, 108, 133, 270, 131, 202, 119, 80, 116, 86, 
                        120, 118, 128, 186, 148, 146, 123, 36, 52]

variant_types_percentages = [2.43, 2.43, 3.99, 3.55, 4.02, 6.81, 3.37, 2.70, 3.17, 3.17, 3.90, 7.92, 3.84,
                             5.93, 3.49, 2.35, 3.40, 2.52, 3.52, 3.46, 3.75, 5.46, 4.34, 4.28, 3.61, 1.06, 1.53]

# 4 segments within each column
variant_types_percentages_phenotypes = [
    [97.59, 2.41, 0, 0], # 5'UTR
    [67.47, 30.12, 2.41, 0], # E1
    [94.12, 5.88, 0, 0], # I1
    [68.6,  28.1, 0, 3.31], # E2
    [89.78, 10.22, 0, 0], # I2
    [61.64, 28.45, 4.74, 5.17], # E3
    [93.91, 6.09, 0, 0], # I3
    [71.74, 20.65, 5.43, 2.17], # E4
    [82.41, 15.74, 1.85, 0], # I4
    [55.56, 36.11, 2.78, 5.56], # E5
    [90.98, 9.02, 0, 0], # I5
    [60, 32.22, 1.85, 5.93], # E6
    [92.37, 6.87, 0.76, 0], # I6
    [57.92, 35.64, 3.47, 2.97], # E7
    [87.39, 11.76, 0.84, 0], # I7
    [61.25, 28.75, 3.75, 6.25], # E8
    [89.66, 10.34, 0, 0], # I8
    [59.3, 23.26, 9.3, 8.14], # E9
    [89.17, 10.83, 0, 0], # I9
    [61.86, 33.9, 4.24, 0], # E10
    [87.5, 10.94, 0.78, 0.78], # I10
    [55.91, 33.87, 4.84, 5.38], # E11
    [89.19, 10.14, 0, 0.68], # I11
    [66.44, 23.97, 6.16, 3.42], # E12
    [89.43, 9.76, 0.81, 0], # I12
    [75, 22.22, 2.78, 0], # E13
    [100, 0, 0, 0] # 3'UTR
]

segment_colors = ["#808080", "#C44E52", "#87CEEB", "#55A868"] # Unknown, cPKU, mPKU, MHP

fig, ax = plt.subplots(figsize=(15, 6))  # Figure size

x = np.arange(len(variant_types))
width = 0.5

# stacked bars
for i in range(4):
    bottom = np.zeros(len(variant_types))

    for j in range(i):
        bottom += np.array(variant_types_counts) * np.array([p[j] for p in variant_types_percentages_phenotypes]) / 100

    values = np.array(variant_types_counts) * np.array([p[i] for p in variant_types_percentages_phenotypes]) / 100

    ax.bar(
        x,
        values,
        width,
        bottom=bottom,
        color=segment_colors[i],
        edgecolor='black',
        linewidth=0.8
    )

# column captions
for i in range(len(variant_types)):
    ax.text(
        i,
        variant_types_counts[i] + max(variant_types_counts) * 0.02,
        f"{variant_types_percentages[i]}%\n{variant_types_counts[i]}",
        ha='center',
        va='bottom',
        fontsize=8 #fontweight='bold'
    )

# scale
ax.set_yscale('linear')

ax.get_yaxis().set_major_formatter(plt.ScalarFormatter())

ax.tick_params(axis='y', labelsize=8)

ax.set_xticks(x)
ax.set_xticklabels(variant_types, fontsize=8)

ax.set_xlabel("Gene region")
ax.set_ylabel("Number of variants")

ax.spines['top'].set_visible(False)
ax.spines['right'].set_visible(False)

ax.yaxis.grid(True, which='both', linestyle='', alpha=0.7)

# Legend
legend_elements = [
    Patch(facecolor=segment_colors[0], edgecolor='black', label='Unknown'),
    Patch(facecolor=segment_colors[1], edgecolor='black', label='cPKU'),
    Patch(facecolor=segment_colors[2], edgecolor='black', label='mPKU'),
    Patch(facecolor=segment_colors[3], edgecolor='black', label='MHP')
]

ax.legend(handles=legend_elements, loc='upper right', frameon=True)

plt.xticks(rotation=0)

plt.tight_layout()
plt.savefig("visualization_results/3_gene_region.png", dpi=300, bbox_inches='tight')
plt.show()
```

### 4. Create a subtable that contains all the data (without NaN or Unknown)¶

In [18]:

```
# subtable raw (with NaN and Unknown)
All_metrics_subtable_raw = PAHvdb_table.iloc[:, [0, 1, 2, 13, 15, 28, 3, 4, 5]]
#All_metrics_subtable_raw

# Clear "Unknown" == 829
All_metrics_subtable_cleared_Unknown = All_metrics_subtable_raw[~All_metrics_subtable_raw.isin(["Unknown"]).any(axis=1)]
#All_metrics_subtable_cleared_Unknown

# Clear APV from "NaN" == 624
All_metrics_subtable_cleared_Unknown_NaN = All_metrics_subtable_cleared_Unknown.dropna()
#All_metrics_subtable_cleared_Unknown_NaN

# Save new table
os.makedirs("results_3", exist_ok=True)

All_metrics_subtable_cleared_Unknown_NaN.to_csv('results_3/PAHvdb_table_All_metrics_subtable_cleared_Unknown_NaN.tsv', sep='\t', index=False)
```

In [19]:

```
All_metrics_subtable_cleared_Unknown_NaN = pd.read_csv("results_3/PAHvdb_table_All_metrics_subtable_cleared_Unknown_NaN.tsv", sep='\t', dtype = str)
#All_metrics_subtable_cleared_Unknown_NaN
```

Out[19]:

|  | PAH Identifier | Protein Variant | cDNA Aberration (NM\_000277.3) | Allelic Phenotype Value (APV) | Allele Frequency (%) | Assigned phenotype | Variant Type | Coding Effect | Gene Region |
| --- | --- | --- | --- | --- | --- | --- | --- | --- | --- |
| 0 | PAH0628 | p.? / p.(Met1Val) | c.1A>G | 0 | 0.029 | cPKU | Substitution | Initiation codon | E1 |
| 1 | PAH0629 | p.? / p.(Met1Leu) | c.1A>T | 0 | 0.01 | cPKU | Substitution | Initiation codon | E1 |
| 2 | PAH0632 | p.? / p.(Met1Arg) | c.2T>G | 0 | 0.01 | cPKU | Substitution | Initiation codon | E1 |
| 3 | PAH1079 | p.? / p.(Met1Thr) | c.2T>C | 0 | 0.029 | cPKU | Substitution | Initiation codon | E1 |
| 4 | PAH0630 | p.? / p.(Met1Ile) | c.3G>A | 0 | 0.006 | cPKU | Substitution | Initiation codon | E1 |
| ... | ... | ... | ... | ... | ... | ... | ... | ... | ... |
| 619 | PAH0699 | p.(Ile65Ser) | c.194T>G | 2.5 | 0.015 | cPKU | Substitution | Missense | E3 |
| 620 | PAH0701 | p.Ile65Thr | c.194T>C | 1.5 | 1.547 | cPKU | Substitution | Missense | E3 |
| 621 | PAH0745 | p.(Glu66Lys) | c.196G>A | 10 | 0.006 | MHP | Substitution | Missense | E3 |
| 622 | PAH0705 | p.(Ser67Pro) | c.199T>C | 3.7 | 0.065 | mPKU | Substitution | Missense | E3 |
| 623 | PAH0610 | p.(=) / IVS12+6T>A | c.1315+6T>A | 5.3 | 0.137 | mPKU | Substitution | Splice Region | I12 |

624 rows × 9 columns

In [20]:

```
# Prints index and column name
for i, col in enumerate(All_metrics_subtable_cleared_Unknown_NaN.columns):
    print(f"Index: {i}, Name: {col}")
```

```
Index: 0, Name: PAH Identifier
Index: 1, Name: Protein Variant
Index: 2, Name: cDNA Aberration (NM_000277.3)
Index: 3, Name: Allelic Phenotype Value (APV)
Index: 4, Name: Allele Frequency (%)
Index: 5, Name: Assigned phenotype
Index: 6, Name: Variant Type
Index: 7, Name: Coding Effect
Index: 8, Name: Gene Region
```

In [25]:

```
# Setting the AF% column values to numbers
All_metrics_subtable_cleared_Unknown_NaN.iloc[:, 4] = pd.to_numeric(All_metrics_subtable_cleared_Unknown_NaN.iloc[:, 4], errors="raise")
```

In [26]:

```
# Sort in descending order
All_metrics_subtable_cleared_Unknown_NaN_AF_sorted = All_metrics_subtable_cleared_Unknown_NaN.sort_values(by=All_metrics_subtable_cleared_Unknown_NaN.columns[4], ascending=False)
All_metrics_subtable_cleared_Unknown_NaN_AF_sorted
```

Out[26]:

|  | PAH Identifier | Protein Variant | cDNA Aberration (NM\_000277.3) | Allelic Phenotype Value (APV) | Allele Frequency (%) | Assigned phenotype | Variant Type | Coding Effect | Gene Region |
| --- | --- | --- | --- | --- | --- | --- | --- | --- | --- |
| 546 | PAH0567 | p.Arg408Trp | c.1222C>T | 0.5 | 16.32 | cPKU | Substitution | Missense | E12 |
| 455 | PAH0468 | p.(=) / IVS10-11G>A | c.1066-11G>A | 0.1 | 6.566 | cPKU | Substitution | Near-splice Region | I10 |
| 317 | PAH0256 | p.Arg261Gln | c.782G>A | 1.5 | 5.183 | cPKU | Substitution | Missense | E7 |
| 286 | PAH0217 | p.Arg243Gln | c.728G>A | 0.4 | 4.613 | cPKU | Substitution | Missense | E7 |
| 573 | PAH0605 | p.(=) / IVS12+1G>A | c.1315+1G>A | 0 | 2.743 | cPKU | Substitution | Splice Donor-Aceptor | I12 |
| ... | ... | ... | ... | ... | ... | ... | ... | ... | ... |
| 111 | PAH1543 | p.(Trp120GlyfsTer75) | c.357del | 0 | 0.002 | cPKU | Deletion | Frame shift | E4 |
| 21 | PAH0862 | p.(Ile25MetfsTer13) | c.75del | 0 | 0.002 | cPKU | Deletion | Frame shift | E2 |
| 537 | PAH0858 | p.(Asn401\_Ser439del) | c.1200-?\_1315+?del | 0 | 0.002 | cPKU | Deletion - Large | In-frame | E12 |
| 109 | PAH1810 | p.(Trp120GlyfsTer75) | c.354del | 0 | 0.002 | cPKU | Deletion | Frame shift | E4 |
| 22 | PAH1012 | p.(Cys29Ter) | c.87C>A | 0 | 0.002 | cPKU | Substitution | Nonsense | E2 |

624 rows × 9 columns

### 4.1 Graph: HIGH frequency variants (AF% > 1%) and their phenotypes¶

In [30]:

```
# subtable Высокая частота (25 variants)
High_frequency_variants = All_metrics_subtable_cleared_Unknown_NaN_AF_sorted[All_metrics_subtable_cleared_Unknown_NaN_AF_sorted.iloc[:, 4] >= 1]
#len(High_frequency_variants)
```

Out[30]:

```
25
```

In [63]:

```
import matplotlib.pyplot as plt

#
PAH_names = list(High_frequency_variants.iloc[:, 0])
Allele_frequences = list(High_frequency_variants.iloc[:, 4])
phenotypes = list(High_frequency_variants.iloc[:, 5])

# Colors for phenotypes
color_map = {
    "cPKU": "#C44E52",
    "mPKU": "#87CEEB",
    "MHP":  "#55A868"
}

# Assign a color to each column based on phenotype
colors = [color_map[p] for p in phenotypes]

# Plotting a graph
plt.figure(figsize=(8, 4))
plt.bar(PAH_names, Allele_frequences, color=colors, edgecolor='black', linewidth=0.8, width=0.5) # Ширина столбцов !


plt.xlabel("PAH ID")
plt.ylabel("Allele frequency (AF%)")
#plt.title("Allele frequencies by PAH variants and phenotypes")

plt.xticks(rotation=90, fontsize=8) #, ha='right'

# legend
legend_elements = [
    Patch(facecolor=color_map["cPKU"], label="cPKU"),
    Patch(facecolor=color_map["mPKU"], label="mPKU"),
    Patch(facecolor=color_map["MHP"], label="MHP"),
]

plt.legend(handles=legend_elements, frameon=True)
plt.tight_layout()

plt.savefig("visualization_results/4_High_frequency_variants.png", dpi=300, bbox_inches='tight')
plt.show()
```

### 4.1 Graph: Variants with AVERAGE frequency (1% > AF% > 0.015%) and their phenotypes¶

In [60]:

```
# subtable High frequency (290 variants)
Medium_frequency_variants = All_metrics_subtable_cleared_Unknown_NaN_AF_sorted[
    (All_metrics_subtable_cleared_Unknown_NaN_AF_sorted.iloc[:, 4] < 1) &
    (All_metrics_subtable_cleared_Unknown_NaN_AF_sorted.iloc[:, 4] >= 0.015)
]
#len(Medium_frequency_variants)
```

Out[60]:

```
290
```

In [61]:

```
import matplotlib.pyplot as plt

# Пример: предполагается, что эти переменные уже заданы
PAH_names = list(Medium_frequency_variants.iloc[:, 0])
Allele_frequences = list(Medium_frequency_variants.iloc[:, 4])
phenotypes = list(Medium_frequency_variants.iloc[:, 5])

# Цвета для фенотипов
color_map = {
    "cPKU": "#C44E52",
    "mPKU": "#87CEEB",
    "MHP":  "#55A868"
}

# Назначаем цвет каждому столбцу по фенотипу
colors = [color_map[p] for p in phenotypes]

# Построение графика
plt.figure(figsize=(10, 5))
plt.bar(PAH_names, Allele_frequences, color=colors, edgecolor='black', linewidth=0.8, width=0.5) # Ширина столбцов !


plt.xlabel("PAH ID")
plt.ylabel("Allele frequency (AF%)")
#plt.title("Allele frequencies by PAH variants and phenotypes")

plt.xticks(rotation=90, fontsize=8) #, ha='right'

# Легенда
legend_elements = [
    Patch(facecolor=color_map["cPKU"], label="cPKU"),
    Patch(facecolor=color_map["mPKU"], label="mPKU"),
    Patch(facecolor=color_map["MHP"], label="MHP"),
]

plt.legend(handles=legend_elements, frameon=True)
plt.tight_layout()

plt.savefig("visualization_results/5_Medium_frequency_variants.png", dpi=300, bbox_inches='tight')
plt.show()
```

### 5. Calculate different values¶

#### 5.1 How many mutations are there in the domains?¶

In [5]:

```
# 5.1 Protein Domain Mutation Location Statistics

value_counts = PAHvdb_table.iloc[:, 6].value_counts()
print("Количество каждого значения:")
print(value_counts)
```

```
Количество каждого значения:
Protein Domain
Not Known          1673
catalytic          1178
regulatory          420
oligomerization     138
Name: count, dtype: int64
```

Protein\_domain = ["Not Known", "catalytic", "regulatory", "oligomerization"]
Protein\_domain\_count = [1673, 1178, 420, 138]
Protein\_domain\_percentages = [49.07, 34.55, 12.32, 4.04]
# Sum = 3409

#### 5.2 How many APV values are available?¶

In [6]:

```
value_counts = PAHvdb_table.iloc[:, 28].value_counts()
print("Количество каждого значения:")
print(value_counts)
```

```
Количество каждого значения:
Assigned phenotype
Unknown    2580
cPKU        680
MHP          75
mPKU         74
Name: count, dtype: int64
```

# Results of counting the number of variants giving assigned phenotypes
phenotype = ["Unknown", "cPKU", "mPKU", "MHP"]
phenotype\_count = [2580, 680, 75, 74]
phenotype\_percentages = [75.68, 19.94, 2.2, 2.17]
# Sum = 3409 (APV = 829)

#### 5.3 How many EA% values are available?¶

In [7]:

```
count_enzyme_activity_1 = PAHvdb_table.iloc[:, 12].count()
print("Сколько вариантов имеют ", count_enzyme_activity_1, sep = "==")

count_enzyme_activity_2 = PAHvdb_table.iloc[:, 12].notna().sum()
print(count_enzyme_activity_2)
```

```
Сколько вариантов имеют ==133
133
```

#### 5.4 How many AF% values are available?¶

In [8]:

```
# 5.4 How many variants have AF% (Allele Frequency (%)) values?

Allele_frequency = PAHvdb_table.iloc[:, 15].count()
print("У скольки вариантов есть значения AF% ?", Allele_frequency, sep = " == ")
```

```
У скольки вариантов есть значения AF% ? == 996
```

#### 5.5 APV vs EA% Correlation¶

In [9]:

```
# 5.5 Correlation between enzyme activity and APV

# Create a new dataframe
Enzemy_APV_table = PAHvdb_table.iloc[:, [1, 2, 12, 13]]
#Enzemy_APV_table

# Replace "-11" with "NaN"
Enzemy_APV_table.iloc[:, [2, 3]] = Enzemy_APV_table.iloc[:, [2, 3]].replace('-11', np.nan)
Enzemy_APV_table
```

```
/tmp/ipykernel_5878/3487995156.py:8: SettingWithCopyWarning: 
A value is trying to be set on a copy of a slice from a DataFrame

See the caveats in the documentation: https://pandas.pydata.org/pandas-docs/stable/user_guide/indexing.html#returning-a-view-versus-a-copy
  Enzemy_APV_table.iloc[:, [2, 3]] = Enzemy_APV_table.iloc[:, [2, 3]].replace('-11', np.nan)
```

Out[9]:

|  | Protein Variant | cDNA Aberration (NM\_000277.3) | Enzyme Activity (%) | Allelic Phenotype Value (APV) |
| --- | --- | --- | --- | --- |
| 1 | p.(=) / -626G>A | c.-626G>A | NaN | NaN |
| 2 | p.(=) / -480\_-478del | c.-480\_-478del | NaN | NaN |
| 3 | p.(=) / -95-385\_-95-382del | c.-95-385\_-95-382del | NaN | NaN |
| 4 | p.(=) / -460del | c.-460del | NaN | NaN |
| 5 | p.(=) / -95-4071\_-95-313del | c.-95-4071\_-95-313del | NaN | NaN |
| ... | ... | ... | ... | ... |
| 3405 | p.(Glu78Val) | c.233A>T | NaN | NaN |
| 3406 | p.(Glu78Asp) | c.234A>T | NaN | NaN |
| 3407 | p.(=) / IVS12+6T>A | c.1315+6T>A | NaN | 5.3 |
| 3408 | p.(=) | c.\*34A>T | NaN | NaN |
| 3409 | p.(=) | c.\*43T>C | NaN | NaN |

3409 rows × 4 columns

In [11]:

```
Enzemy_APV_table.dropna(inplace=True)
Enzemy_APV_table.to_csv('results_3/Enzemy_APV_table.tsv', sep='\t', index=False)
Enzemy_APV_table
```

```
/tmp/ipykernel_5878/443589024.py:1: SettingWithCopyWarning: 
A value is trying to be set on a copy of a slice from a DataFrame

See the caveats in the documentation: https://pandas.pydata.org/pandas-docs/stable/user_guide/indexing.html#returning-a-view-versus-a-copy
  Enzemy_APV_table.dropna(inplace=True)
```

Out[11]:

|  | Protein Variant | cDNA Aberration (NM\_000277.3) | Enzyme Activity (%) | Allelic Phenotype Value (APV) |
| --- | --- | --- | --- | --- |
| 341 | p.Phe39Leu | c.117C>G | 49 | 0.2 |
| 346 | p.Leu41Phe | c.121C>T | 10 | 0 |
| 384 | p.Arg53His | c.158G>A | 79 | 9.4 |
| 524 | p.(=) / IVS2-13T>G | c.169-13T>G | 6.7 | 0 |
| 577 | p.Arg68Gly | c.202A>G | 40 | 5 |
| ... | ... | ... | ... | ... |
| 3372 | p.Gly46Ser | c.136G>A | 16 | 1.6 |
| 3376 | p.Ala47Val | c.140C>T | 123 | 10 |
| 3379 | p.Leu48Ser | c.143T>C | 39 | 1.8 |
| 3393 | p.(Ile65Ser) | c.194T>G | 32 | 2.5 |
| 3395 | p.Ile65Thr | c.194T>C | 33 | 1.5 |

99 rows × 4 columns

### With EA% = 123% и 111%¶

In [3]:

```
# Saved, removed 111
Enzemy_APV_table = pd.read_csv("Enzemy_APV_table_more100.tsv", sep='\t', dtype = str)
#Enzemy_APV_table

Enzemy_APV_table.iloc[:, 2] = pd.to_numeric(Enzemy_APV_table.iloc[:, 2], errors='coerce')
Enzemy_APV_table.iloc[:, 3] = pd.to_numeric(Enzemy_APV_table.iloc[:, 3], errors='coerce')
```

In [5]:

```
# The Spearman's rank correlation coefficient
from scipy.stats import spearmanr
import numpy as np
import pandas as pd

# 3-й и 4-й столбцы (индексы 2 и 3)
x = list(np.array(Enzemy_APV_table.iloc[:, 2]))
y = list(np.array(Enzemy_APV_table.iloc[:, 3]))

rho, p_value = spearmanr(x, y) #

print(f"Коэффициент Спирмена: {rho}")
print(f"p-value: {p_value}") # p-value = 1.906e-18
```

```
Коэффициент Спирмена: 0.7924391076330781
p-value: 1.5118858822127594e-22
```

### Without EA% = 123% и 111%¶

In [7]:

```
# Saved, removed 111
Enzemy_APV_table = pd.read_csv("Enzemy_APV_table_no_more100.tsv", sep='\t', dtype = str)
#Enzemy_APV_table

Enzemy_APV_table.iloc[:, 2] = pd.to_numeric(Enzemy_APV_table.iloc[:, 2], errors='coerce')
Enzemy_APV_table.iloc[:, 3] = pd.to_numeric(Enzemy_APV_table.iloc[:, 3], errors='coerce')
```

In [8]:

```
# The Spearman's rank correlation coefficient
from scipy.stats import spearmanr
import numpy as np
import pandas as pd

# 3-й и 4-й столбцы (индексы 2 и 3)
x = list(np.array(Enzemy_APV_table.iloc[:, 2]))
y = list(np.array(Enzemy_APV_table.iloc[:, 3]))

rho, p_value = spearmanr(x, y) #

print(f"Коэффициент Спирмена: {rho}")
print(f"p-value: {p_value}") # p-value = 1.906e-18
```

```
Коэффициент Спирмена: 0.7958558492433815
p-value: 2.0344445817317146e-22
```

In [ ]:
